# Performance and Agreement When Annotating Chest X-ray Text Reports—A Preliminary Step in the Development of a Deep Learning-Based Prioritization and Detection System

**DOI:** 10.3390/diagnostics13061070

**Published:** 2023-03-11

**Authors:** Dana Li, Lea Marie Pehrson, Rasmus Bonnevie, Marco Fraccaro, Jakob Thrane, Lea Tøttrup, Carsten Ammitzbøl Lauridsen, Sedrah Butt Balaganeshan, Jelena Jankovic, Tobias Thostrup Andersen, Alyas Mayar, Kristoffer Lindskov Hansen, Jonathan Frederik Carlsen, Sune Darkner, Michael Bachmann Nielsen

**Affiliations:** 1 Department of Diagnostic Radiology, Copenhagen University Hospital, Rigshospitalet, 2100 Copenhagen, Denmark; 2 Department of Clinical Medicine, University of Copenhagen, 2100 Copenhagen, Denmark; 3 Department of Computer Science, University of Copenhagen, 2100 Copenhagen, Denmark; 4 Unumed Aps, 1055 Copenhagen, Denmark; 5 Radiography Education, University College Copenhagen, 2200 Copenhagen, Denmark; 6 Novo Nordisk Foundation Center for Protein Research, Faculty of Health and Medical Sciences, University of Copenhagen, 2100 Copenhagen, Denmark; 7 Department of Health Sciences, Panum Institute, University of Copenhagen, 2100 Copenhagen, Denmark

**Keywords:** chest X-ray, deep learning, artificial intelligence, agreement, performance, text annotation, data, radiologists, development

## Abstract

A chest X-ray report is a communicative tool and can be used as data for developing artificial intelligence-based decision support systems. For both, consistent understanding and labeling is important. Our aim was to investigate how readers would comprehend and annotate 200 chest X-ray reports. Reports written between 1 January 2015 and 11 March 2022 were selected based on search words. Annotators included three board-certified radiologists, two trained radiologists (physicians), two radiographers (radiological technicians), a non-radiological physician, and a medical student. Consensus labels by two or more of the experienced radiologists were considered “gold standard”. Matthew’s correlation coefficient (MCC) was calculated to assess annotation performance, and descriptive statistics were used to assess agreement between individual annotators and labels. The intermediate radiologist had the best correlation to “gold standard” (MCC 0.77). This was followed by the novice radiologist and medical student (MCC 0.71 for both), the novice radiographer (MCC 0.65), non-radiological physician (MCC 0.64), and experienced radiographer (MCC 0.57). Our findings showed that for developing an artificial intelligence-based support system, if trained radiologists are not available, annotations from non-radiological annotators with basic and general knowledge may be more aligned with radiologists compared to annotations from sub-specialized medical staff, if their sub-specialization is outside of diagnostic radiology.

## 1. Introduction

Chest X-rays (CXRs) are the most commonly performed diagnostic image modality [1]. Recent technological advancements have made it possible to create systems that support and increase radiologists’ efficiency and accuracy when analyzing CXR images [2]. Thus, interest in developing artificial intelligence-based systems for detection and prioritization of CXR findings has increased, including how to efficiently gather training data [3].

For training, validating, and testing a deep learning algorithm, labeled data are required [4]. Previous ontological schemes have been developed to have consistent labeling. Labeling schemes can vary, from hierarchical labeling systems with 180+ unique labels [5] to few selected labels [6,7]. Label creation for deep learning development may be unique to each project, since they are dependent on factors such as imaging modality, body part, algorithm type, etc. [4]. In a previous study we developed a labeling scheme for annotation of findings in CXRs to obtain consistent labeling [8]. Our labeling scheme was tested for inter- and intra-observer agreement when used to annotate CXR images [8], and iterations have been ongoing to potentially increase consistent use of labels for annotation of CXR image and text reports. 

Optimally, CXR training data should consist of manually labeled findings on the radiographic images, marked with e.g., bounding boxes for location, and radiologists are often needed to perform such a task to ensure the most accurate labeling [9]. Gathering data for training an algorithm may therefore be time-consuming and expensive. Several systems for automatic extraction of labels from CXR text reports have therefore been developed, including natural language processing models based on either feature engineering [6,10] or deep learning technology [11]. Labels that are extracted this way can then be linked to the corresponding CXR image to provide large, labeled image datasets using minimal time and cost [5]. 

To fully automate the labeling process, researchers have attempted to develop unsupervised machine learning engineering to extract labels [12]. However, these methods still seem inferior compared to solutions with components of supervision [13,14]. Therefore, just as with images, text labeling algorithms still need manually labeled data for training. 

Labeling of text for training a deep learning algorithm needs to be consistent [15]. However, unlike images, labeling and annotation of text may not require specialized radiologists, since radiological reports are used for communication with other specialty fields in health care and therefore should be understood by a much more diverse group of people than just radiologists [16]. Only a few studies have been done on reading comprehension and understanding findings in radiological text reports, when readers are health care workers with differentiated levels of radiological experience [17]. Understanding how variability in radiological knowledge impacts reading comprehension of a radiological text report, could not only be beneficial in the development of a deep learning algorithm but could also give insight to pitfalls of a radiological text report as a communicative tool between medical staff [18]. 

In this study we aimed to investigate how differentiated levels of radiological task experience impact reading comprehension and labeling performance on CXR text reports. We also field-tested the text report labeling scheme by measuring label-specific agreement between predicted and actual labels as to decrease any potential bias to reading comprehension created by the labeling process itself. 

## 2. Materials and Methods

Ethical approval was obtained on 11 May 2022 by the Regional Council for Region Hovedstaden (R-22017450). Approval for data retrieval and storage was obtained on 19 May 2022 by the Knowledge Center on Data Protection Compliance (P-2022-231).

### 2.1. Diagnostic Labeling Scheme for Text Annotations

**Figure 1:**
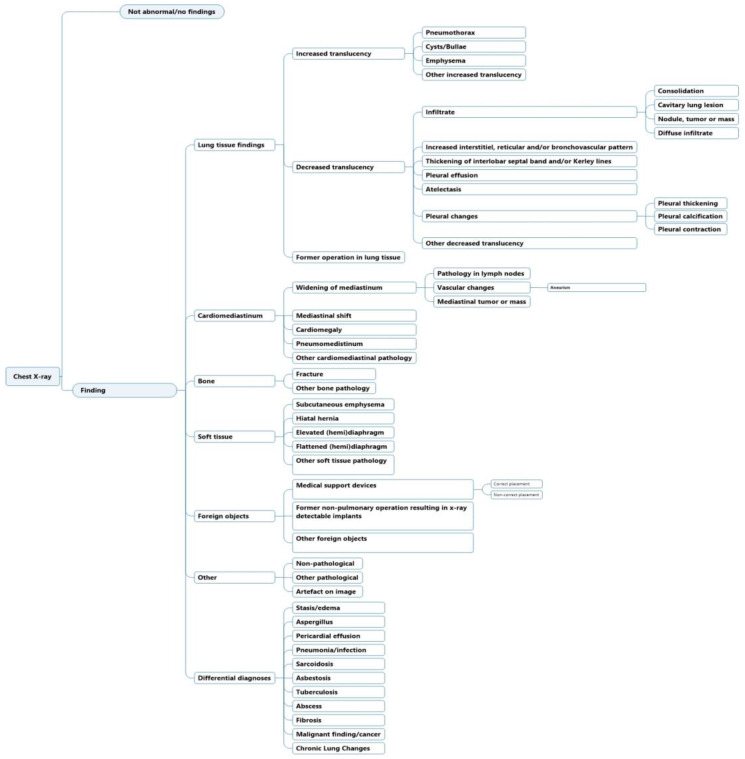
Labeling hierarchy for chest X-ray text report annotation.

The initial structure and development of the labeling scheme have previously been highlighted [8]. In summary, the labels were generated to match existing CXR ontologies such as Fleischner criteria and definitions [19] and other machine learning labeling schemes [5,6,7]. Labels were ordered hierarchically, where a high-level class such as “decreased translucency” was divided to lower-level classes that increased in specificity. The labeling scheme was previously tested for inter- and intra-observer agreement in CXR image annotation [8]. Iterations were since made to increase the agreement; (1) labels were made to be as descriptive as possible and (2) interpretive labels were added under the category “Differential diagnosis”, because of increased detailed information that was present in chest X-ray text reports compared to chest X-ray images (Figure 1).

### 2.2. Dataset

A selection of a total of 200 de-anonymized CXR reports from 1 January 2015 to 11 March 2022 were collected at the Department of Diagnostic Radiology at Rigshospitalet through the PACS system (AGFA Impax Client 6, Mortsel, Belgium). The CXR reports were retrieved through two methods:

Firstly, through a computerized search algorithm, CXR reports were selected using search words found in the text. A minimum of six CXR reports were required to be present for each of the following search words; pneumothorax, cysts/bullae, emphysema, infiltrate, consolidation, diffuse infiltrate, pleural effusion, atelectasis, lung surgery, chronic lung changes, pneumonia infection, tuberculosis, abscess, and stasis/edema. This method resulted in 84 reports.

Secondly, for the remaining 116 reports, a computerized search algorithm was used to find and distribute an equal number of cases, between the following criteria (29 cases each):

(1) Truly randomly selected.
(2) Randomly selected cases containing any abnormal findings.
(3) Randomly selected cases, within the top 10% of all cases that had the greatest number of associated labels per case relative to the length of the report.
(4) Randomly selected cases, within the bottom 10% of cases that had the least number of labels associated per case relative to the length of the report.

### 2.3. Participants and Annotation Process

A total of three board-certified radiologists were included as annotators to determine labels for the cases in the text annotation set to form the “gold standard” labels (actual labels). All three radiologists had specialized training ranging from 14 to 30+ years each. Six annotators with varying degrees of radiological experience were included to annotate the 200 text reports with labels from the labeling scheme (Figure 1). Annotators included a(n): intermediate radiologist (physician with radiological experience, 6 years), novice radiologist (physician with radiological experience, 2 years), experienced radiographer (radiological technician, with radiographer experience of 15 years), novice radiographer (radiological technician with radiographer experience of 3 years), non-radiological physician (7 years of other specialized, clinical experience, post-graduation), and a senior medical student (planning to graduate from university within 6 months).

**Figure 2:**
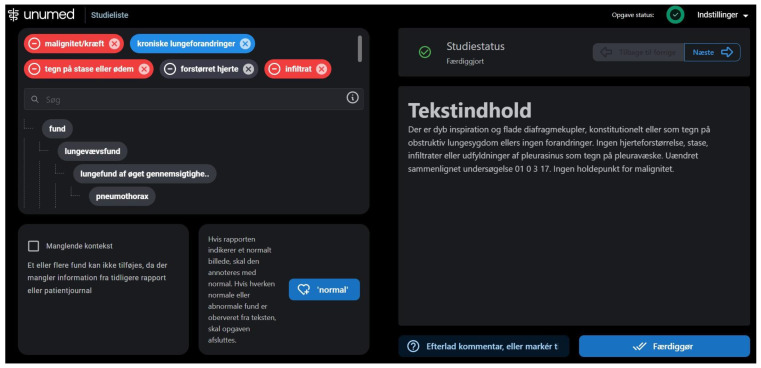
Annotation software for text report annotations. The full-text report is displayed on the right side and labels in the labeling hierarchy are displayed on the left. On the top left, selected labels are showcased; red labels for negative findings and blue labels for positive findings.

The annotation process began on 25 August 2022, and ended on 25 October 2022. All 200 text reports were imported to a proprietary annotation software developed by Unumed Aps (Copenhagen, Denmark). Annotators were instructed to find and label each piece of text describing both positive and negative findings (Figure 2). Annotators were blinded to the X-ray images and other annotators’ annotations.

### 2.4. Presentation of Data and Statistical Analysis

“Gold standard” labels were defined as consensus on a label in a text report between two or more of the three board-certified radiologists. “Majority” vote labels were defined by consensus on a label between four or more of the six annotators and “majority excl. intermediate radiologist” were defined as consensus vote on a label between three or more of the remaining annotators after removing the intermediate radiologist as an annotator. Frequency counts reflected the total cumulative counts of a label’s use in all text reports in the annotation set. Time spent on annotation was done by calculating the average time spent on a text report from opening the report to annotation completion.

Matthew’s correlation coefficient (MCC) [20] was used to compare annotator performance to “gold standard” labeling and to compare annotators’ performance to each other. The MCC was based on values selected for a 2 × 2 confusion matrix (Table 1) where true positive (TP) described the number of labels that matched “gold standard” labels for all positive and negative findings separately. True negative (TN) described the number of labels that were not used by annotators which also matched labels that were not used by both “gold standard” for all positive and negative findings separately. False positives (FP) described the number of labels that annotators used, but “gold standard” did not use, and false negative (FN) described all labels that “gold standard” used but annotators did not use.

MCC was then defined by following equation [20]:

$$MCC=\frac{\left(TP*TN\right)-\left(FP*FN\right)}{\sqrt{\left(TP+FP\right)\left(TP+FN\right)\left(TN+FP\right)\left(TN+FN\right)}}$$

To achieve this, MCC was calculated using Python 3.8.10 (https://www.python.org/) with the Pandas [21] and Numpy [22] libraries for each label and then micro-averaged [23] to give an overall coefficient for all positive and negative labels. MCC ranges between −1 and 1, where 1 represents perfect positive correlation, 0 represents correlation not better than random, and −1 represents total disagreement between labels of the “gold standard” set (actual) and the set of labels chosen by the annotator (predicted) [20].

One weakness of MCC and other standard agreement statistics is that they fail to take partial agreement into account in structured and taxonomic annotation tasks like ours. In addition, they do not clearly identify tendencies towards over- or under-annotation by any single annotator. To this end, we performed a separate analysis for any pair of annotators. An annotator here means either an individual human annotator or a constructed annotator such as “gold standard” or any of the “majority”-categories. For each annotator pair, we ran a maximum weight matching algorithm on a graph constructed from their individual annotations, trying to pair the labels from the two annotators as best as possible. We used the implementation available in the Python library networkx (version 2.8.8) [24].

We employed a weighting that enforced the following criteria in descending order:

(1) Match with the exact same label, or
(2) Match with an ancestral or descendent node (e.g., for “vascular changes” it could be either “aneurism” or “widening of mediastinum” etc. (Figure 1))

The hierarchical order in which the labels are placed, categorizes labels into similar groups and findings of similar characterization become more distinguishable from each other with each branch division. This is done to reduce the number of unusable labels caused by inter-reader variability [25] as disagreement on a label in a branched division could have common ascending nodes. Annotators do not manually mark a piece of text to a label, so to maximize data, we post-processed by discarding matched pairs of labels that did not belong to the same branch, since we operated on the assumption that the same piece of text/finding should not lead to annotation with labels that did not belong within the same category. The statistical algorithm would pair up any remaining annotations at random after all matches with positive weight had been made. If the annotators made an unequal number of annotations, such that it was impossible to pair all annotations, or if matched labels did not belong within the same branch or were not in a direct line of descending/ascending order we denoted the remaining annotations as unmatched.

Descriptive statistics were thus calculated to investigate specific agreements by comparing counts of “matched” and “unmatched” labels between annotators and “gold standard”. In addition to presenting matched and unmatched labels as representation for individual annotator agreements, the number of matched and unmatched counts was also presented for each label.

## 3. Results

**Figure 3:**
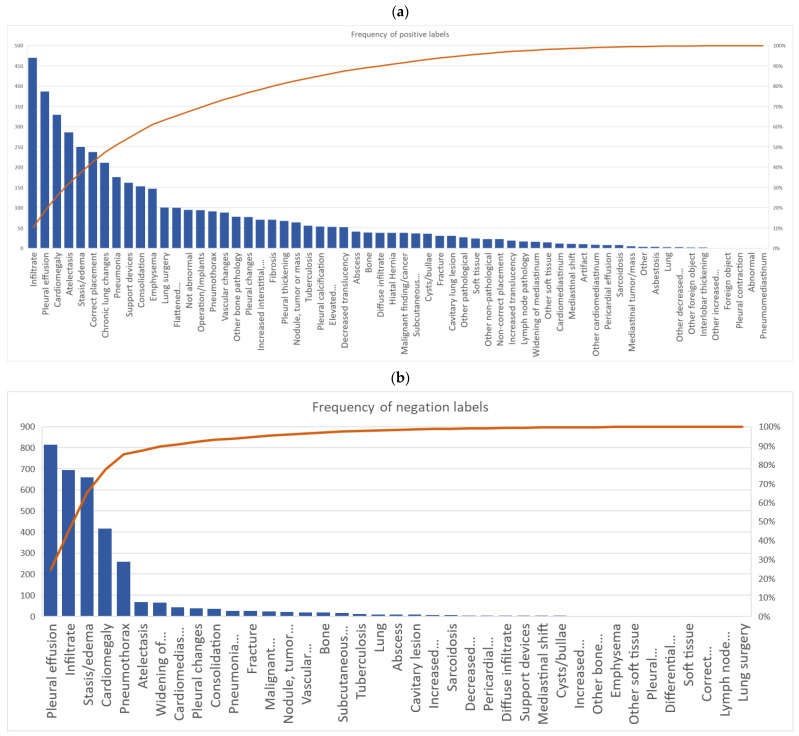
Pareto chart of all annotators accumulated use of labels for (**a**) positive findings and (**b**) negative findings.

A total of 63 positive labels and 62 negative labels were possible to use for annotation (Figure 1). A pareto chart showed that 25 labels covered 80% of all labeled positive findings, and four labels covered 80% of all negative findings. The top 5 most used labels for positive findings were: “infiltrate”, “pleural effusion”, “cardiomegaly”, “atelectasis”, and “stasis/edema”. The top 5 most used labels for negative findings were: “pleural effusion”, “infiltrate”, “stasis/edema”, “cardiomegaly”, and “pneumothorax” (Figure 3a,b).

For labels that represented positive findings, the novice radiographer had more annotations for “bone” (16 cases vs. 0–8 cases) and “decreased translucency” (29 cases vs. 0–10 cases) compared to other annotators. The novice radiologist had more annotations for “other non-pathological” compared to other annotators (18 cases vs. 0–2 cases), and the senior medical student had more annotations on “diffuse infiltrate” compared to other annotators (22 cases vs. 0–5 cases) (Table A1 in Appendix A).

For negative findings, the experienced radiographer had more annotations on “consolidation” (23 cases vs. 0–4 cases) and “pleural changes” (20 cases vs. 0–6 cases) compared to the other annotators. The non-radiological physician had more annotations on “cardiomediastinum” than other annotators (21 cases vs. 0–7 cases) (Table A2 in Appendix A).

The average time spent on annotating a text report was: 98.1 s for the intermediate radiologist, 76.2 s for the novice radiologist, 232.1 s for the experienced radiographer, 135 s for the novice radiographer, 99.4 s for the non-radiological physician, 145.8 s for the senior medical student, and each “gold standard” annotator took on average 135.2 s per text report.

### 3.1. Annotator Performance and Agreement

Table 2a,b showed the MCC values for each annotator for positive and negative findings, respectively. The intermediate radiologist had the best MCC compared to other annotators, both for labels representing positive findings and negative findings (MCC 0.77 and MCC 0.92). The senior medical student had comparable MCC values to the novice radiologist for both negative and positive findings (Table 2a,b).

For both positive and negative findings, the senior medical student achieved better MCC than the non-radiological physician (0.71 vs. 0.64 for positive findings and 0.88 vs. 0.77 for negative findings). This tendency was also present for the radiographers. The novice radiographer achieved better MCC for both positive and negative findings compared to the experienced radiographer (0.65 vs. 0.57 for positive findings and 0.88 vs. 0.64 for negative findings).

All annotators achieved higher MCC for negative findings compared to their own MCC for positive findings (Table 2a,b).

The number of labels that were a match (Table 3) and unmatched (Table A3) between different pairs of annotators was used as representation for degree of agreement between different annotators.

Table 3 showed the number of matched labels between each annotator for both positive and negative findings. The intermediate radiologist, novice radiologists and senior medical student had the most label matches with each other. The novice radiographer had more matches with the “gold standard” (710 labels matched) compared with the experienced radiographer’s matches with “gold standard” (589 labels matched). The senior medical student had more matches with “gold standard” (741 labels matched) compared with the non-radiological physician’s matches with “gold standard” (665 labels matched).

Table A3 in the Appendix A showed the number of unmatched labels that were left after subtracting the number of matched labels to each annotator’s total label use. The intermediate radiologist had the least number of unmatched labels left compared with the “gold standard” (201), however, the other annotators closely followed (203–234). The “majority” vote achieved the lowest number of unmatched labels against “gold standard” annotations compared with any individual annotator (122). “Gold standard” generally used fewer labels per text report compared with any annotator. (e.g., 32 unmatched labels leftover for “gold standard” when matched to the intermediate radiologist vs. 201 unmatched labels leftover for the intermediate radiologist when matched to “gold standard”).

The “majority excl. the intermediate radiologist” voting (723) had more labels that matched with “gold standard” compared with the “majority” voting which included the intermediate radiologist (702) (Table 3). Even though the number of unmatched labels increased (162) when excluding the intermediate radiologist majority vote compared with majority voting including the intermediate radiologist (122), there were still fewer unmatched labels than any individual annotator (Table A3).

### 3.2. Label Specific Agreement

Table 4 and Table 5 showed the cumulative cases of matches on a specific label for labels in the “lung tissue findings” category and “cardiomediastinum” category, respectively. “Atelectasis”, “infiltrate”, and “pleural effusion” were lung tissue related labels with the most matches (219, 687, and 743, respectively) (Table 4), while “cardiomegaly” (472) was the label with the most matches in the “cardiomediastinum” category (Table 5), and “medical device, correct placement” (115), and “stasis/edema” (576) were the labels with the most matches in the rest of the labeling scheme (Table A4).

For the label “infiltrate”, the annotators had a greater spread across different labels compared to “gold standard”. When “gold standard” used the label “infiltrate”, annotators matched with six labels other than “infiltrate”. Four of these labels were more specific i.e., descendants of “infiltrate” and two were less specific i.e., ancestors of “infiltrate” (Figure 1 and Table 4). For comparison, “gold standard” matched only with two descendent labels and one ancestral label (Table 4).

The opposite tendency was seen in the labels “decreased translucency”, “pleural changes”, and “atelectasis”—“gold standard” had greater spread and used more specific labels compared to annotators (Table 4).

**Table 5:** Number of matched cases (accumulated) on specific labels in the labeling scheme related to “cardiomediastinal findings”. * Rows and columns not belonging to the parent node “cardiomediastinal findings” and that did not have any label disagreements have been pruned and thus number of rows does not match number of columns.

	Gold Standard *						
Annotators *		Cardiomediastinum	Cardiomegaly	Widening of Mediastinum	Lymph Node Pathology	Other Cardiomediastinum	Vascular Changes
	Cardiomediastinum	1	16	8	1	1	
	Cardiomegaly	3	472				
	Widening of Mediastinum	1		36	1		
	Lymph node pathology				9		
	Mediastinal tumor			1			
	Other cardiomediastinum					4	
	Vascular changes			2			32
		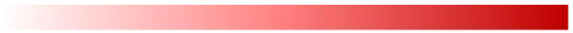 0 labels matched 100+ labels matched					

When annotators used “cardiomediastinum” it was most often matched with more specific, descendent nodes such as “cardiomegaly”, “widening of mediastinum”, and “lymph node pathology” by “gold standard” (Table 5). Annotators were also less specific when “gold standard” used “lymph node pathology” since annotators only matched with using ancestral nodes besides the label itself (Table 5).

For the rest of the labeling scheme “gold standard” also used more specific labels compared to annotators (Table A4).

For unmatched labels, annotators had more different types of unmatched labels compared to “gold standard” (60 different types of labels vs. 41). Annotators had labeled 760 findings that were unmatched with “gold standard” labels, while “gold standard” only had 131 findings that did not find a match within the annotators’ labels.

## 4. Discussion

There were three main findings in our study: (1) for radiologists, annotation performance of CXR text reports increased when radiological experience increased, (2) annotators had better performance on annotating negative findings compared to positive findings, and (3) annotators with less radiological experience tended to use a greater amount of less specific labels compared to experienced radiologists.

### 4.1. Performance of Annotators

Generally, all annotators showed high correlation [20] to “gold standard” annotations of CXR text reports (Table 2a,b). This finding was comparable to a previous study which showed a similar level of agreement between radiologists and non-radiological physicians and medical students when reading and comprehending radiology reports [26]. However, disagreements in reading and reporting radiological findings exist even between readers of the same specialty [27]. Previous studies suggested that the free-form structure of a radiological text report permitted the use of sentences that were ambiguous and inconsistent [28]. The variability in using these phrases could contribute to the annotation variability observed between the annotators. The intermediate radiologist’s specialized experience may enable them to be better aligned with the “gold standard” annotators in interpreting whether an ambiguously worded sentence suggested that a finding was relevant and/or important enough to be annotated [26,29].

Our study also showed that the senior medical student and the novice radiographer performed better in annotation than the non-radiological physician and the experienced radiographer, respectively (Table 2a,b). Previous studies have demonstrated the difference between adaptive and routine expertise [30]. Experienced medical staff are encouraged to increase their specialization over time, thus, narrowing, but deepening their field of knowledge and therefore do not often engage in unknown situations [31,32], contrary to younger medical staff in active training. The novice radiographer and the medical student may have been more receptive to the change in their usual tasks, making them quicker to adapt to the annotation process itself [33,34]. The inherent routine expertise the experienced radiographer and the non-radiological physician have, may affect their behavior to value efficiency higher than thoroughness [35,36], and to only annotate findings that they would usually find relevant and disregard other findings [26,37]. A previous study aligned with our findings and showed that radiologists in training had slightly better performance compared to sub-specialist radiologists when reading and understanding reports outside their sub-specialty [38]. Another study showed that clinicians extract information from a radiological report based on their clinical bias [39,40] which may also contribute to the result of lesser correlation with “gold standard” annotations by the non-radiological physician compared to e.g., the senior medical student.

We found that labeling negative findings or labeling normal cases from abnormal cases may result in more consistent data for training a decision support system. Our findings were congruent with previous findings where it was demonstrated that negative findings were described more unambiguously in text reports, and that this may contribute to less difficulty in reading and comprehending negative findings compared to positive findings [27]. Negations may be a useful resource in the development of artificial intelligence-based algorithms for radiological decision support systems and studies [10,41,42] have shown that they are just as crucial to identify in a text, as positive findings [43].

### 4.2. Majority Vote Labeling

The results of our research indicated that there could be a reduction in false positive labels when using majority labeling compared to the labels used by an individual annotator (Table A3). Recent efforts have been made to outsource labeling to more annotators of lesser specialized experience as a way to reduce the time and cost of data gathering compared to sourcing and reimbursing field experts in the same tasks [44]. Several methods have been proposed to clean data labeled by multiple, less experienced annotators to obtain high-quality datasets efficiently, including using majority-vote labeling [45,46,47]. More inexperienced annotators may tend to overinterpret and overuse labels due to lack of training [48] or fear of missing findings [49]. Our study suggested that using majority labeling instead of using labels by individual annotators may eliminate some of the noisy and dispensable labels created by inexperienced annotators. Even when we eliminated the most experienced annotator from the majority voting (intermediate radiologist), there was still a reduction in false positive labels compared to any individual annotator (Table A3).

### 4.3. The Labeling Scheme

“Atelectasis”, “infiltrate”, “pleural effusion”, “cardiomegaly”, “correctly placed medical device”, and “stasis/edema” were the labels that were most frequently agreed upon from our labeling scheme (Table 4, Table 5 and Table A4 in Appendix A). While some labeling taxonomies are highly detailed with more labels than our labeling scheme [5], our labels were comparable to previously used annotation taxonomies which used text mining methods to extract labels [6,50]. An increased number of labels may introduce noise in data gathering [51], which there is a particularly high risk of when interpreting CXR and thoracic findings [52]. Fewer and broader labels may therefore be more desirable since this may enable higher agreement on a label from different readers.

Although “infiltrate” was one of the most agreed-upon labels, the differential diagnosis “pneumonia/infection” was not, despite it being one of the most common referral reasons for a CXR [53]. The “pneumonia/infection” diagnosis is usually based on a combination of clinical and paraclinical findings [54]. Radiologists are aware of this and may oftentimes not be conclusive in their reports, thus, introducing larger uncertainty to words associated with “pneumonia” compared to “infiltrate” [52]. Comparable with previous results from labeling CXR images [8], our study suggested that labels which are descriptive may be preferred to interpretive diagnostic labels. When annotating CXR reports, uncertainty of the radiologist in making diagnostic conclusions may introduce increased annotation bias in text reports.

### 4.4. Bias, Limitations and Future Studies

Due to time constraints, only a limited number of CXR text reports were included in our study. Previous studies have mentioned the limitations of using Cohen’s kappa when it comes to imbalanced datasets, specifically, when the distribution of true positives and true negatives is highly skewed [55]. The limitations have been shown to be most prevalent when readers show negative or no correlation [56]. In anticipation of a label imbalance in our dataset and a risk of none to negative correlation between an annotator and “gold standard”, we used Matthew’s correlation coefficient over Cohen’s kappa. However, as shown by Chicco et al. [56] MCC and Cohen’s kappa are closely related, especially when readers show positive correlation. In our study, all readers had positive correlation coefficients with “gold standard” and the interpretation of results would therefore likely not have changed if we had used Cohen’s kappa instead of MCC.

A limitation of the number of annotators included in our study was due to a combination of time constraints and participant availability. We recognize that as with the “gold standard” labels, ideally each level of annotator-experience should consist of multiple annotators’ consensus vote. However, we found it relevant that our study reflected the real-world obstacles of data-gathering for deep learning development projects since recruitment of human annotators is already a well-known problem. We presented “majority” voting categories as solutions to, not only the limited number of annotators in our study, but also as a solution when there is a lack of annotators in deep learning development projects in general.

Annotations by the board-certified experienced radiologists may not reflect true labels, since factors such as the annotation software and subjective opinions may influence a radiologist’s annotations. We attempted to reduce these elements of reader bias through consensus between the experienced radiologists by majority voting [57]. Furthermore, since annotators did not manually link each specific text piece to a label, we could not guarantee that annotators labeled the exact same findings with the same labels. We used an algorithm for matching labels in this study, since that algorithm would also be used for developing the final artificial intelligence-based support system.

Our study did not investigate whether an artificial intelligence-based algorithm would perform better when trained on annotations from less experienced medical staff compared to experienced radiologists. The assumption behind our study was that radiologists could provide annotations of the highest quality to train an algorithm, and that annotators with higher correlation to those annotations would produce high quality data [9]. Further studies are needed to investigate the differences in algorithm performance based on training data annotated by experienced radiologists compared to other medical staff. We did not investigate whether our annotators’ text report labels corresponded to the CXR image, since this was not within the scope of our study but could be a topic of interest for future studies.

## 5. Conclusions

Trained radiologists were most aligned with experienced radiologists in understanding a chest X-ray report. For the purpose of labeling text reports for the development of an artificial intelligence-based decision support system, performance increased with radiological experience for trained radiologists. However, as annotators, medical staff with general and basic knowledge may be preferred to experienced medical staff, if the experienced medical staff have sub-specialized routine experience in other domains than diagnosing thoracic radiological findings.

## Figures and Tables

**Table 1:** An example of 2 × 2 confusion matrix for the calculations of Matthew’s Correlation Coefficient. TP, true positive; FP, false positive; FN, false negative; TN, true negative.

	Gold Standard		
Annotator(s)		Labels used	Labels NOT used
	Labels used	TP	FP
	Labels NOT used	FN	TN

**Table 2:** Matthew’s correlation coefficients (MCC) for annotators’ performance in annotating chest X-ray text reports compared to gold standard annotation set for (**a**) positive findings and (**b**) negative findings.

	Radiologist, Intermediate	Radiologist, Novice	Radiographer, Experienced	Radiographer, Novice	Physician, Non-Radiologist	Senior Medical Student
**MCC**	0.77	0.71	0.57	0.65	0.64	0.71
(**a**)						
	**Radiologist, Intermediate**	**Radiologist, Novice**	**Radiographer, Experienced**	**Radiographer, Novice**	**Physician, Non-Radiologist**	**Senior Medical Student**
**MCC**	0.92	0.88	0.64	0.88	0.77	0.88
(**b**)						

**Table 3:** Number of matched labels of both positive and negative findings for each annotator, majority of annotators, and gold standard.

	Radiologist, Intermediate	Radiologist, Novice	Radiographer, Experienced	Radiographer, Novice	Physician, Non-Radiologist	Senior Medical Student	Majority	Majority excl. Intermed. Radiologist	Gold Standard
Radiologist, intermediate		849	679	785	753	832	794	810	766
Radiologist, novice	849		654	763	744	811	779	815	740
Radiographer, experienced	679	654		642	597	669	664	680	589
Radiographer, novice	785	763	642		710	791	753	801	710
Physician, non-radiologist	753	744	597	710		741	714	746	665
Senior medical student	832	811	669	791	741		783	823	741
Majority	794	779	664	753	714	783		824	702
Majority excl. Intermed. Radiologist	810	815	680	801	746	823	824		723
Gold Standard	766	740	589	710	665	741	702	723	
	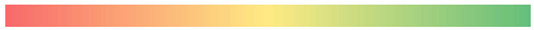 Fewest matched (worst) 50% fractile Most matched (best)								

**Table 4:** Number of matched cases (accumulated) on specific labels in the labeling scheme related to “lung tissue findings”. * Rows and columns not belonging to the parent node “lung tissue findings” and that did not have any label disagreements have been pruned and thus number of rows does not match number of columns.

	Gold Standard *											
Annotators *		Atelectasis	Consolidation	Cysts/Bullae	Increased Interstitial	Infiltrate	Decreased Translucency	Nodule, Tumor or Mass	Pleural Calcification	Pleural Changes	Pleural Effusion	Pleural Thickening
	Atelectasis	219										
	Cavitary lesion					8						
	Consolidation		22			53	3					
	Cysts/bullae			21								
	Diffuse infiltrate					30						
	Increased interstitial…				20							
	Infiltrate		4			687	1	10				
	Lung	1		1		1					2	
	Decreased translucency	1	3		1	5	5	2	2	1	9	1
	Nodule, tumor or mass					8		28				
	Pleural calcification								32			
	Pleural changes								10	10		13
	Pleural effusion										743	
	Pleural thickening											30
		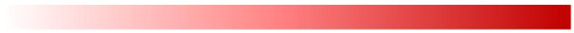 0 labels matched 100+ labels matched										

## Data Availability

Not applicable.

## Appendix A

**Table A1:** Frequency counts of labels used by each annotator for positive findings.

		Annotators									
		Radiologist, Inter- Mediate	Radiologist, Novice	Radiographer, Experienced	Radiographer, Novice	Physician, Non-Radiologist	Senior Medical Student	Senior Radiologist 3	Senior Radiologist 2	Senior Radiologist 1	All
**Labels**	Abnormal	0	0	1	0	0	0	0	0	0	1
	Abscess	6	5	5	3	5	5	5	6	1	41
	Asbestosis	0	1	1	0	1	0	0	0	1	4
	Atelectasis	33	31	31	32	33	33	35	35	23	286
	Bone	0	6	0	16	5	3	8	1	0	39
	Cardiomediastinum	0	0	0	1	9	0	1	1	0	12
	Cardiomegaly	39	37	38	38	34	36	36	39	33	330
	Cavitary lesion	2	2	8	6	3	4	2	0	4	31
	Chronic lung changes	33	33	17	28	17	29	21	26	7	211
	Consolidation	25	29	33	5	12	25	12	7	5	153
	Correct placement	32	42	11	29	34	7	27	24	32	238
	Cysts/bullae	4	2	7	4	4	3	4	4	4	36
	Decreased translucency	3	0	0	29	10	3	4	3	0	52
	Diffuse infiltrate	3	5	0	0	22	3	0	5	0	38
	Elevated (hemi)diaphragm	7	7	7	6	6	6	6	6	2	53
	Emphysema	16	15	18	24	19	18	10	10	17	147
	Enlarged mediastinum	2	2	2	2	0	2	2	2	2	16
	Fibrosis	12	10	11	8	10	7	7	4	2	71
	Flattened (hemi)diaphragm	8	13	14	13	12	13	15	10	2	100
	Foreign object	0	0	0	1	0	0	0	0	0	1
	Fracture	7	1	4	0	1	4	3	4	7	31
	Hiatal hernia	5	5	4	3	3	5	4	5	4	38
	Increased interstitial....	6	10	5	1	5	16	3	11	14	71
	Increased translucency	1	0	1	2	13	1	0	1	0	19
	Infiltrate	52	45	53	64	39	62	60	31	64	470
	Interlobar septal thickening	0	1	1	0	0	0	0	0	0	2
	Lung	0	0	0	0	1	2	0	0	0	3
	Lung surgery	5	12	7	17	15	19	5	14	7	101
	Lymph node pathology	2	3	2	2	0	2	2	3	1	17
	Malignant/cancer	3	3	4	5	7	7	2	7	0	38
	Mediastinal shift	2	3	1	0	0	1	1	2	1	11
	Mediastinal tumor	0	1	2	0	0	1	0	0	1	5
	Nodule, tumor or mass	6	8	3	4	10	6	8	3	16	64
	Not abnormal	9	23	13	2	16	10	12	6	4	95
	Non-correct placement	2	2	3	4	1	1	3	6	1	23
	Operation/implants	23	10	8	1	6	3	17	10	16	94
	Artifact	0	7	2	0	0	0	0	1	0	10
	Other bone pathology	11	9	7	5	12	11	5	12	6	78
	Other cardiomediastinum	1	1	0	0	1	3	1	1	1	9
	Other	1	0	0	0	0	0	0	3	0	4
	Other foreign object	0	0	0	1	0	0	1	0	0	2
	Other decreased translucency	1	0	0	0	1	0	0	1	0	3
	Other increased translucency	0	0	0	0	1	0	0	0	0	1
	Other non-pathological	1	18	2	0	0	0	0	2	0	23
	Other pathological	8	1	11	0	2	3	1	1	0	27
	Other soft tissue	0	0	1	1	3	5	1	1	3	15
	Pericardial effusion	1	0	0	1	1	1	3	1	0	8
	Pleural calcification	8	8	1	2	8	7	5	8	7	54
	Pleural changes	11	6	13	13	11	8	7	4	4	77
	Pleural contraction	0	0	1	0	0	0	0	0	0	1
	Pleural effusion	41	43	42	38	41	47	49	44	42	387
	Pleural thickening	10	10	0	5	8	6	7	10	12	68
	Pneumomediastinum	0	0	1	0	0	0	0	0	0	1
	Pneumonia	32	32	19	18	29	14	0	30	2	176
	Pneumothorax	10	10	10	10	13	10	10	10	8	91
	Sarcoidosis	1	1	1	1	1	1	0	1	1	8
	Soft tissue	0	1	0	8	11	0	4	0	0	24
	Stasis/edema	30	31	23	23	32	26	29	29	27	250
	Subcutaneous emphysema	6	6	4	0	5	5	2	5	3	37
	Support devices	10	0	34	40	12	12	11	17	3	162
	Tuberculosis	8	8	3	6	8	8	6	6	8	56
	Vascular changes	15	0	15	11	0	0	16	11	0	88

**Table A2:** Frequency counts of labels used by each annotator for negative findings.

		Annotators									
		Radiologist, Inter-Mediate	Radiologist, Novice	Radiographer, Experienced	Radiographer, Novice	Physician, Non-Radiologist	Senior Medical Student	Senior Radiologist 3	Senior Radiologist 2	Senior Radiologist 1	All
**Labels**	Abscess	1	1	1	1	1	1	1	1	0	8
	Atelectasis	9	8	8	10	6	8	5	8	7	69
	Bone	3	3	0	3	1	2	4	2	0	18
	Cardiomediastinum	0	7	1	0	21	5	5	5	0	44
	Cardiomegaly	52	43	50	53	31	48	43	42	55	417
	Cavitary lesion	0	0	3	2	0	1	1	0	1	8
	Consolidation	2	1	23	1	0	4	1	1	2	35
	Correct placement	0	0	1	0	0	0	0	0	0	1
	Cysts/bullae	0	0	3	0	0	0	0	0	0	3
	Differential diagnosis	0	0	1	0	0	0	0	0	0	1
	Decreased translucency	0	0	0	1	3	0	0	1	0	5
	Diffuse infiltrate	0	0	2	0	1	0	0	1	0	4
	Emphysema	0	0	0	1	0	0	1	0	0	2
	Enlarged mediastinum	18	9	2	8	0	6	11	11	1	66
	Fracture	3	4	3	1	3	3	3	3	3	26
	Increased interstitial	1	0	1	1	0	1	1	1	1	7
	Increased translucency	0	0	0	1	1	0	0	0	0	2
	Infiltrate	86	73	60	84	65	88	81	77	82	696
	Lung	2	1	0	0	0	5	0	0	0	8
	Lung surgery	0	0	0	0	0	0	0	1	0	1
	Lymph node pathology	0	0	0	0	0	0	1	0	0	1
	Malignant/cancer	3	3	5	3	4	2	2	2	0	24
	Mediastinal shift	1	1	1	0	0	0	0	0	0	3
	Nodule, tumor or mass	1	9	1	1	2	2	1	1	4	22
	Other bone pathology	0	0	0	1	1	0	0	0	0	2
	Other soft tissue	0	0	0	1	0	0	0	0	0	1
	Pericardial effusion	0	0	2	1	0	0	2	0	0	5
	Pleural changes	1	2	20	0	6	3	2	1	4	39
	Pleural effusion	102	102	44	94	81	102	99	97	94	815
	Pleural thickening	0	0	1	0	0	0	0	0	0	1
	Pneumonia	8	7	3	0	1	1	0	7	0	27
	Pneumothorax	30	31	28	29	26	30	30	30	25	259
	Sarcoidosis	1	1	0	0	1	1	0	1	1	6
	Soft tissue	0	0	0	0	0	0	1	0	0	1
	Stasis/edema	82	81	51	80	63	77	76	78	72	660
	Subcutaneous emphysema	2	2	2	0	2	2	1	2	2	15
	Support devices	0	0	1	1	0	0	0	2	0	4
	Tuberculosis	2	2	0	0	1	1	0	2	2	10
	Vascular changes	3	0	0	2	0	10	4	0	0	19

**Table A3:** Number of unmatched labels of both positive and negative findings after subtraction of matched labels by individual annotators, majority of annotators, and gold standard annotations.

		Number of Unmatched Labels (by Annotator)								
		Radiologist, Intermediate	Radiologist, Novice	Radiogra-pher, Experienced	Radiogra-pher, Novice	Physician, Non-Radiologist	Senior Medical Student	Majority	Majority excl. Intermed. Radiologist	Gold Standard
**Compared to annotator**	Radiologist, intermediate		101	144	128	121	114	30	75	32
	Radiologist, novice	118		169	150	130	135	45	70	58
	Radiographer, experienced	288	296		271	277	277	180	205	209
	Radiographer, novice	182	187	181		164	155	71	84	88
	Physician, non-radiologist	214	206	226	203		205	110	139	133
	Senior medical student	135	139	154	122	133		41	62	57
	Majority	173	171	179	160	160	163		61	96
	Majority excl. Intermed. Radiologist	157	135	143	112	128	123	0		75
	Gold Standard	201	210	234	203	209	205	122	162	
		Fewest unmatched (best) 50% fractile Most unmatched (worst)								
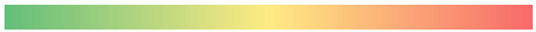

**Table A4:** Number of matched cases (accumulated) on specific labels in the labeling scheme for all labels except labels in the “lung tissue findings” category and the “cardiomediastinum” category. * Rows and columns belonging to the parent nodes “lung tissue finding” or “cardiomediastinal findings” and that did not have any label disagreements have been pruned and thus number of rows does not match number of columns.

	Gold Standard *									
**Annotators ***		Bone	Correct Placement	Fracture	Non-Correct Placement	Operation and Implants	Other Bone Pathology	Stasis/Edema	Subcutaneous Emphysema	Support Devices
	Bone	11		10			10			
	Correct placement		115							13
	Differential diagnosis							1		
	Foreign object					1				
	Fracture	2		29						
	Non-correct placement				6					1
	Operation and implants					38				
	Other bone pathology	3					38			
	Soft tissue								1	
	Stasis/edema							576		
	Subcutaneous emphysema								28	
	Support devices		48							24
		0 labels matched 100+ labels matched								
